# Whole-body patterns of the range of joint motion in young adults: masculine type and feminine type

**DOI:** 10.1186/s40101-016-0112-8

**Published:** 2016-10-01

**Authors:** Keiichi Moromizato, Ryosuke Kimura, Hitoshi Fukase, Kyoko Yamaguchi, Hajime Ishida

**Affiliations:** 1 Department of Human Biology and Anatomy, Graduate School of Medicine, University of the Ryukyus, Uehara 207, Nishihara, Okinawa 903-0215 Japan; 2 Division of Human Evolution Studies, Graduate School of Medicine, Hokkaido University, Kita 15 Nishi 7, Kita-ku, Sapporo, Hokkaido 060-8638 Japan; 3 Present address: School of Natural Sciences and Psychology, Liverpool John Moores University, Byrom Street, Liverpool, L3 3AF UK

**Keywords:** Range of motion, Joints, Young adult, Principal component analysis, Multiple regression analysis, Sexual dimorphism, Hand/foot dominance

## Abstract

**Background:**

Understanding the whole-body patterns of joint flexibility and their related biological and physical factors contributes not only to clinical assessments but also to the fields of human factors and ergonomics. In this study, ranges of motion (ROMs) at limb and trunk joints of young adults were analysed to understand covariation patterns of different joint motions and to identify factors associated with the variation in ROM.

**Methods:**

Seventy-eight healthy volunteers (42 males and 36 females) living on Okinawa Island, Japan, were recruited. Passive ROM was measured at multiple joints through the whole body (31 measurements) including the left and right side limbs and trunk.

**Results:**

Comparisons between males and females, dominant and non-dominant sides, and antagonistic motions indicated that body structures influence ROMs. In principal component analysis (PCA) on the ROM data, the first principal component (PC1) represented the sex difference and a similar covariation pattern appeared in the analysis within each sex. Multiple regression analysis showed that this component was associated with sex, age, body fat %, iliospinale height, and leg extension strength.

**Conclusions:**

The present study identified that there is a spectrum of “masculine” and “feminine” types in the whole-body patterns of joint flexibility. This study also suggested that body proportion and composition, muscle mass and strength, and possibly skeletal structures partly explain such patterns. These results would be important to understand individual variation in susceptibility to joint injuries and diseases and in one’s suitable and effective postures and motions.

**Electronic supplementary material:**

The online version of this article (doi:10.1186/s40101-016-0112-8) contains supplementary material, which is available to authorized users.

## Introduction

In the field of orthopaedics and rehabilitation medicine, measuring range of motion (ROM) is a clinical procedure to evaluate a mechanical joint problem caused by disorders of the locomotor apparatus. The purpose of ROM measurement is not only to observe the extent of inhibition but also to identify the factors that restrict joint movement and to evaluate the effectiveness of treatment and training. A measurement method of ROM was established by the American Academy of Orthopaedic Surgeons (AAOS) [1], in which the standard anatomical position was defined as the neutral zero starting position, and this method has been used internationally. The AAOS has provided the reference values for normal joint ROM. However, there is great variation in ROM even among healthy individuals, depending on sex, age, physical constitution, daily activities etc. Therefore, in clinical assessments using ROM, it is important to establish an individual standard for each patient. For this purpose, it is indispensable to identify which biological and physical factors affect ROM. In addition, understanding the correlations in ROM of different joints can improve clinical assessments for each individual.

There have been many studies of joint ROM to date. Most of the studies have focused only on upper or lower extremity joint motions, focusing on the effects of age, sex, and/or side dominance and on some special population such as sports athletes and disease patients [2–9]. Even in the studies that have examined six major limb joints of the upper and lower limbs [10–18], correlations among different joints have not been sufficiently discussed. In addition, there have been few studies measuring ROM throughout the whole body, including the trunk joints [19].

Understanding whole-body patterns of joint flexibility and identifying their related biological and physical factors contribute not only to clinical assessments but also to the fields of human factors and ergonomics. Biological and physical factors such as age, sex, physical constitution, and daily activities can affect patterns of joint flexibility. Then, whole-body patterns of joint flexibility can have influences on whole-body motions and eventually can be important to know one’s suitable and effective postures and motions.

The aims of this study were to understand the whole-body patterns of joint flexibility and to identify factors associated with variations in ROM. For this purpose, we measured ROMs of the limb and trunk joints of young adults and analysed their association with biological and physical factors. We also compared dominant and non-dominant sides to obtain a cue for related factors. Principal component analysis (PCA) was performed using multiple joint ROM data to identify covariation patterns among different joints. It was found that a major pattern that explains the variation between sexes also appears within each sex, and this pattern appears to be associated with some somatometric and sthenometric measurements.

## Materials and methods

### Subjects

We recruited volunteers living in Okinawa Island, Japan. Inclusive criteria were healthy males and females between the ages of 20 and 29 who did not have any joint diseases and any history of orthopaedic surgery on joints. As shown in Additional file 1: Table S1, the subjects consisted of 36 females and 42 males. Their ages were concentrated in early twenties and ranged from 20 to 25 years in females (mean 20.8 years, SD 1.2 years) and from 20 to 29 years in males (mean 21.4 years, SD 1.9 years). Hand/arm and foot/leg dominances were determined based on a questionnaire [20, 21]. Most individuals had an experience of sports when they were high school students. There was no significant difference in the frequencies of sports experience between males and females (Fisher’s exact test). All subjects provided their written informed consent to participate in this research project.

### Measurements

ROM data were collected from the subjects using a goniometer (OG Giken. Co. Ltd., Okayama, Japan). All ROM measurements were performed by four observers after confirming that the inter-observer errors in the measurements were small (0.97 < ICC < 0.99). The motions examined are shown in Table 1.

**Table 1:** Summary of somatometry and ROM for each joint motion

Item	All		Female		Male	
	Mean	SD	Mean	SD	Mean	SD
Height [cm]	163.7	9.0	156.9	5.2	168.9	7.7
Weight [kg]	57.0	9.3	51.8	6.4	61.1	9.3
Body fat percentage [%]	20.6	5.2	22.1	3.3	19.4	6.1
Lean body mass [kg]	45.3	8.0	40.3	5.0	49.2	7.8
ROMs [°]						
Shoulder flexion	176.4	5.6	178.4	3.2	174.7	6.5
Shoulder extension	66.5	6.2	67.6	7.1	65.7	5.4
Shoulder abduction	179.7	1.0	179.6	0.9	179.8	1.2
Shoulder external rotation	92.9	9.3	94.5	8.2	91.5	10.1
Shoulder internal rotation	62.5	11.0	67.4	9.2	58.4	10.9
Shoulder horizontal flexion	133.6	9.4	137.1	9.2	130.7	8.6
Shoulder horizontal extension	56.1	8.6	58.3	8.5	54.3	8.3
Elbow flexion	142.6	4.9	144.5	4.0	141.0	5.1
Elbow extension	4.3	5.4	5.6	6.1	3.2	4.6
Wrist extension	81.1	9.1	83.7	7.9	79.0	9.6
Wrist flexion	88.2	10.8	89.6	12.9	87.0	8.5
Fingers V MCP flexion	104.9	12.9	104.6	14.6	105.2	11.4
Fingers V MCP extension	71.8	15.7	73.6	14.1	70.3	16.8
Trunk flexion	35.3	9.5	32.9	9.3	37.3	9.2
Trunk extension	28.2	8.7	28.3	7.6	28.2	9.7
Trunk rotation	48.3	10.8	43.9	11.3	52.2	8.8
Trunk lateral bending	23.2	4.6	21.3	4.2	24.8	4.4
Hip flexion	128.4	6.7	130.4	7.8	126.7	5.1
Hip extension	17.0	3.9	16.1	3.7	17.9	3.9
Hip abduction	33.0	4.8	34.1	5.3	32.1	4.1
Hip adduction	13.8	6.3	13.9	4.1	13.7	7.7
Hip external rotation	43.9	8.4	40.6	8.1	46.7	7.6
Hip internal rotation	42.1	9.6	47.9	9.3	37.1	6.5
Knee flexion	147.9	5.9	148.9	5.0	147.1	6.4
Knee extension	3.5	4.8	5.3	5.4	2.0	3.7
Ankle dorsi flexion	23.0	6.5	23.9	6.2	22.3	6.8
Ankle plantar flexion	51.9	6.6	54.1	6.6	50.1	6.1

The average of dominant and non-dominant is shown

Height, weight, upper limb length, iliospinale height, forearm circumference, forearm minimum circumference, calf circumference, and ankle circumference were measured following the standard anthropometric method [22]. On the digitally scanned images of the left hand, the second and fourth finger lengths were measured using software, ImageJ (ImageJ, NIH, Bethesda, MD, USA), and the 2D:4D ratio was calculated.

Grip strength was measured using the Smedley Hand Dynamometer (OG Giken. Co. Ltd). In the measurements, the subject was in a standing position with arms at their side, not touching their body. We obtained the average of three trials. As for leg strength, the flexor and extensor muscle strengths at the knee were measured using the Biodex System 3 dynamometer (Sakaimed Co. Ltd., Tokyo, Japan). Isometric knee flexion and extension strengths were tested at 60° of knee flexion. Peak torque was recorded for each motion in three trials and the average peak torque was calculated.

The calliper method was used to calculate body fat percentage (BF%). Subcutaneous fat thickness was measured at the mid-point of the posterior surface of an upper arm, at the inferior angle point of the scapula, and at the side of the lateral point from the umbilicus. Then, BF% and lean body mass (LBM) was calculated using the following formula [23–25]:

$$ \mathrm{Body}\;\mathrm{surface}\;\mathrm{area}\;\left({\mathrm{cm}}^2\right):\kern0.37em S = 72.46 \times {H}^{0.725} \times {W}^{0.425}, $$

$$ \mathrm{Body}\;\mathrm{density}:\;D = 1.0935 - 0.000297 \times T \times S\ /\ W\ /\ 100, $$

$$ \mathrm{B}\mathrm{F}\% = \left(4.570\ /\ D - 4.142\right) \times 100, $$

$$ \mathrm{L}\mathrm{B}\mathrm{M} = W \times \left(100 - \mathrm{B}\mathrm{F}\%\right)/100, $$

where *W*, *H*, and *T* are weight (kg), height (cm), and the sum of subcutaneous fat thicknesses (mm), respectively. The results of the somatometric and sthenometric measurements are shown in Table 1 and Additional file 1: Table S2, respectively.

### Statistical analyses

Statistical analyses were performed using SPSS® Statistics version 19 (IBM Japan, Tokyo, Japan) and Excel Statistics (Social Survey Research Information Co. Ltd., Tokyo, Japan). Basic summary statistics were calculated for each sex. To identify biological and physical factors associated with each ROM, multiple regression analysis was used. Differences between dominant and non-dominant sides were examined by paired *t* test, in which we subtracted ROM for dominant side from ROM for non-dominant side (ROM(ND) − ROM(D)). PCA was performed to elucidate whole-body patterns of joint ROM. Correlation coefficient and partial correlation coefficient controlling for sex were calculated between each principal component (PC) and each factor. Finally, to disclose factors associated with the whole-body patterns of ROM, multiple regression analysis was conducted. As for bilateral measurements, the averages of the left and right sides were input into these statistical analyses except for the test of the side difference.

## Results

### Effects of sex, age, height, BF%, and LBM on each motion

The results of measurements are summarized in Table 1. To concretely explain the factors responsible for the individual variation in ROM, multiple regression analyses were performed for each motion, including sex, age, body height, BF%, and LBM as explanatory variables. The female sex significantly increased ROMs for shoulder flexion, internal rotation and horizontal flexion, elbow flexion and extension, wrist extension, and hip flexion, adduction, and internal rotation, but decreased ROMs for hip extension and external rotation, and trunk flexion and rotation (Table 2). Hip extension versus flexion and hip external rotation versus internal rotation are pairs of antagonistic motions. When the total ranges of these antagonistic motions were compared, no significant sex difference was found (data not shown).

**Table 2:** Multiple regression analysis for identifying factors associated with ROM for each joint motion

Dependent variable	Sex (F:0, M:1)			Age			Height			Body fat %			Lean body mass					
	*B*	*β*	*P*	*B*	*β*	*P*	*B*	*β*	*P*	*B*	*β*	*P*	*B*	*β*	*P*	Constant	*P*	*R* ^2^
Shoulder flexion	−4.68	−0.42	*2.5E*−*02*	−0.05	−0.02	8.9E−01	−0.03	−0.05	8.0E−01	0.04	0.04	7.5E−01	0.14	0.20	3.6E−01	178.39	*3.8E*−*13*	0.13
Shoulder extension	−4.16	−0.33	7.7E−02	0.73	0.19	1.0E−01	0.00	0.00	9.9E−01	0.22	0.18	1.5E−01	0.23	0.30	1.7E−01	37.97	9.9E−02	0.12
Shoulder abduction	0.51	0.24	2.1E−01	0.08	0.13	2.8E−01	−0.02	−0.21	3.4E−01	−0.01	−0.06	6.2E−01	−0.01	−0.07	7.6E−01	182.46	*4.8E*−*55*	0.06
Shoulder external rotation	−0.90	−0.05	7.8E−01	−1.67	−0.29	*8.7E*−*03*	0.36	0.34	9.5E−02	−0.46	−0.25	*3.6E*−*02*	−0.66	−0.57	*5.6E*−*03*	109.72	*9.2E*−*04*	0.23
Shoulder internal rotation	−13.06	−0.59	*1.2E*−*03*	−0.16	−0.02	8.3E−01	−0.05	−0.04	8.4E−01	0.13	0.06	6.2E−01	0.46	0.33	1.0E−01	57.62	1.3E−01	0.21
Shoulder horizontal flexion	−11.41	−0.61	*2.9E*−*04*	−1.75	−0.30	*3.2E*−*03*	0.38	0.37	5.2E−02	−0.56	−0.30	*5.9E*−*03*	−0.13	−0.11	5.4E−01	130.98	*3.1E*−*05*	0.34
Shoulder horizontal extension	−1.14	−0.07	7.1E−01	−0.68	−0.13	2.5E−01	0.42	0.44	*3.9E*−*02*	0.16	0.10	4.3E−01	−0.62	−0.59	*5.8E*−*03*	27.60	3.6E−01	0.18
Elbow flexion	−5.62	−0.57	*1.4E*−*03*	0.69	0.22	*3.7E*−*02*	0.17	0.31	1.3E−01	−0.26	−0.27	*2.2E*−*02*	−0.14	−0.23	2.6E−01	115.15	*1.5E*−*09*	0.25
Elbow extension	−4.67	−0.43	*1.5E*−*02*	−0.95	−0.28	*9.4E*−*03*	0.16	0.27	1.8E−01	−0.34	−0.32	*7.5E*−*03*	−0.07	−0.10	6.0E−01	9.93	5.9E−01	0.24
Wrist extension	−8.09	−0.44	*9.7E*−*03*	−2.21	−0.39	*3.0E*−*04*	0.26	0.26	1.9E−01	−0.32	−0.18	1.2E−01	−0.02	−0.01	9.4E−01	96.62	*1.8E*−*03*	0.29
Wrist flexion	−6.92	−0.32	6.0E−02	−2.69	−0.40	*2.3E*−*04*	−0.12	−0.10	6.3E−01	−0.18	−0.09	4.5E−01	0.57	0.43	*3.2E*−*02*	145.25	*1.2E*−*04*	0.27
Fingers V MCP flexion	−1.50	−0.06	7.7E−01	−0.88	−0.11	3.6E−01	0.15	0.11	6.4E−01	−0.05	−0.02	8.7E−01	0.03	0.02	9.3E−01	98.48	*4.9E*−*02*	0.02
Fingers V MCP extension	−2.54	−0.08	6.7E−01	0.53	0.06	6.4E−01	−0.56	−0.32	1.6E−01	0.41	0.13	3.0E−01	0.68	0.35	1.1E−01	113.74	5.6E−02	0.06
Trunk flexion	8.19	0.43	*1.4E*−*02*	1.83	0.31	*4.0E*−*03*	−0.03	−0.03	8.8E−01	0.52	0.28	*1.6E*−*02*	−0.18	−0.16	4.2E−01	−4.52	8.9E−01	0.25
Trunk extension	−1.98	−0.11	5.6E−01	−0.18	−0.03	7.9E−01	0.29	0.30	2.0E−01	−0.17	−0.10	4.5E−01	−0.23	−0.21	3.4E−01	−0.17	1.0E + 00	0.03
Trunk rotation	13.78	0.64	*1.9E*−*04*	1.66	0.25	*1.5E*−*02*	0.03	0.03	8.9E−01	0.62	0.29	*9.4E*−*03*	−0.36	−0.27	1.6E−01	4.34	9.0E−01	0.33
Trunk lateral bending	3.03	0.33	7.8E−02	−0.11	−0.04	7.3E−01	0.00	0.01	9.7E−01	0.04	0.04	7.3E−01	0.05	0.09	6.7E−01	20.00	2.3E−01	0.15
Hip flexion	−6.13	−0.46	*1.7E*−*02*	0.46	0.11	3.4E−01	0.20	0.27	2.2E−01	−0.10	−0.07	5.6E−01	−0.08	−0.10	6.4E−01	94.77	*2.6E*−*04*	0.11
Hip extension	3.34	0.43	*2.1E*−*02*	0.41	0.17	1.3E−01	−0.03	−0.07	7.5E−01	0.20	0.27	*3.2E*−*02*	−0.04	−0.09	6.6E−01	9.24	5.1E−01	0.16
Hip abduction	−2.88	−0.30	1.1E−01	−0.46	−0.16	1.8E−01	−0.07	−0.12	5.8E−01	−0.21	−0.22	7.4E−02	0.09	0.16	4.6E−01	55.00	*2.4E*−*03*	0.13
Hip adduction	−4.68	−0.37	*4.6E*−*02*	0.13	0.03	7.6E−01	−0.04	−0.06	7.8E−01	0.16	0.13	3.0E−01	0.47	0.61	*4.9E*−*03*	−4.57	8.4E−01	0.16
Hip external rotation	10.18	0.61	*4.4E*−*04*	0.83	0.16	1.2E−01	0.03	0.03	8.6E−01	0.65	0.39	*7.1E*−*04*	−0.19	−0.19	3.3E−01	11.53	6.7E−01	0.31
Hip internal rotation	−11.45	−0.60	*4.4E*−*04*	0.45	0.08	4.5E−01	−0.03	−0.03	8.7E−01	−0.15	−0.08	4.8E−01	0.02	0.02	9.3E−01	46.37	1.3E−01	0.33
Knee flexion	−3.51	−0.30	1.2E−01	0.48	0.13	2.6E−01	0.26	0.41	7.3E−02	−0.12	−0.11	4.0E−01	−0.22	−0.31	1.6E−01	109.06	*4.0E*−*06*	0.09
Knee extension	−3.09	−0.32	8.2E−02	−0.38	−0.13	2.6E−01	−0.14	−0.27	2.1E−01	−0.06	−0.06	6.3E−01	0.14	0.24	2.5E−01	31.16	7.3E−02	0.16
Ankle dorsi flexion	0.02	0.00	9.9E−01	0.43	0.11	3.6E−01	0.17	0.24	2.9E−01	0.05	0.04	7.5E−01	−0.34	−0.42	5.7E−02	0.27	9.9E−01	0.09
Ankle plantar flexion	−3.64	−0.28	1.4E−01	−0.49	−0.12	3.0E−01	0.16	0.22	3.1E−01	0.07	0.05	6.6E−01	−0.17	−0.21	3.4E−01	43.67	7.5E−02	0.12

*P* values less than 0.05 are shown in italics

*B* partial regression coefficient, *β* standardized partial regression coefficient

The multiple regression analyses also showed that older age is significantly associated with lower ROMs for shoulder external rotation and horizontal flexion, elbow extension, wrist flexion and extension, and higher ROMs for elbow flexion and trunk flexion and rotation.

A higher LBM was significantly related with lower ROMs for shoulder external rotation and horizontal extension, and with higher ROMs for wrist flexion and hip adduction (Table 2). BF% negatively affected ROMs for shoulder external rotation, shoulder horizontal flexion, and elbow flexion and extension. In contrast, BF% was positively associated with trunk flexion and rotation, and hip extension and external rotation.

### ROM differences between dominant and non-dominant sides

Significant ROM differences between dominant and non-dominant sides were detected for several motions. The non-dominant side had higher mobility than the dominant side for shoulder internal rotation, hip abduction, and ankle plantar flexion, whereas the opposite was observed for shoulder external rotation, wrist flexion, and hip adduction (Table 3). In the total range of antagonistic motions, however, there were no significant differences except for ankle dorsiflexion and plantarflexion in all the subjects (Table 4).

**Table 3:** Subtraction of the dominant from the non-dominant side, ROM(ND) − ROM(D) [°]

Body part	ROM	All			Female			Male		
		Mean	SD	*P*	Mean	SD	*P*	Mean	SD	*P*
Upper	Shoulder flexion	1.0	5.0	1.0E−01	0.3	2.7	5.4E−01	1.6	6.5	1.4E−01
	Shoulder extension	0.5	6.9	5.5E−01	0.6	6.5	6.0E−01	0.4	7.3	7.4E−01
	Shoulder abduction	0.3	1.4	1.0E−01	0.4	1.9	1.8E−01	0.1	0.8	3.2E−01
	Shoulder external rotation	−1.6	6.3	*3.2E−02*	−1.3	7.0	2.8E−01	−1.9	5.6	*4.6E−02*
	Shoulder internal rotation	4.3	17.0	*3.5E−02*	5.1	15.9	6.8E−02	3.6	18.1	2.4E−01
	Shoulder horizontal flexion	−0.5	9.0	6.5E−01	−0.9	7.7	5.1E−01	−0.1	10.1	9.4E−01
	Shoulder horizontal extension	−0.5	9.8	6.7E−01	−0.3	9.5	8.6E−01	−0.7	10.1	6.9E−01
	Elbow flexion	1.1	5.6	9.2E−02	1.2	4.9	1.7E−01	1.1	6.1	2.9E−01
	Elbow extension	0.2	2.6	5.0E−01	−0.3	3.0	5.7E−01	0.7	2.1	5.8E−02
	Wrist extension	1.2	8.2	2.2E−01	0.4	5.7	6.5E−01	1.9	10.0	2.6E−01
	Wrist flexion	−2.0	7.7	*3.4E−02*	−1.0	7.8	4.4E−01	−2.8	7.6	*2.9E−02*
	Fingers V MCP flexion	0.7	10.3	5.6E−01	2.2	9.4	1.8E−01	−0.7	10.9	7.1E−01
	Fingers V MCP extension	0.6	10.7	6.5E−01	0.6	11.3	7.8E−01	0.6	10.2	7.3E−01
Trunk	Trunk rotation	0.3	6.6	7.2E−01	−1.0	5.5	2.9E−01	1.4	7.4	2.4E−01
	Trunk lateral bending	0.3	4.1	4.9E−01	0.1	4.3	8.4E−01	0.5	4.1	4.4E−01
Lower	Hip flexion	−0.5	7.4	5.8E−01	−1.0	7.7	4.4E−01	0.0	7.2	1.0E + 00
	Hip extension	0.8	3.5	5.1E−02	0.6	3.2	2.9E−01	1.0	3.8	1.0E−01
	Hip abduction	3.5	8.1	*3.7E−04*	4.3	9.2	*9.7E−03*	2.8	6.9	*1.6E−02*
	Hip adduction	−3.2	10.6	*1.1E−02*	−2.0	5.3	*3.3E−02*	−4.3	13.7	5.7E−02
	Hip external rotation	2.0	9.8	8.0E−02	3.4	8.4	*2.1E−02*	0.8	10.8	6.6E−01
	Hip internal rotation	−0.5	8.7	6.0E−01	−1.4	9.9	4.0E−01	0.3	7.5	8.3E−01
	Knee flexion	0.7	6.1	3.5E−01	−1.0	6.0	3.3E−01	2.1	5.9	*2.8E−02*
	Knee extension	0.3	2.5	3.6E−01	0.4	2.8	4.0E−01	0.2	2.3	6.8E−01
	Ankle dorsi flexion	1.5	7.1	7.8E−02	2.0	5.8	5.1E−02	1.0	8.1	4.4E−01
	Ankle plantar flexion	2.1	7.0	*1.2E−02*	1.7	6.2	1.1E−01	2.4	7.7	5.8E−02

Non-dominant (ND) and dominant (D) sides were determined by hand/arm for upper body and by foot/leg for lower body. *P* values less than 0.05 are shown in italics

**Table 4:** Subtraction of the dominant from the non-dominant side in the total range of antagonistic motions, ROM(ND) − ROM(D) [°]

Body part	Antagonistic motions	All			Female			Male		
		Mean	SD	*P*	Mean	SD	*P*	Mean	SD	*P*
Upper	Shoulder flx + ext	1.5	9.3	1.8E−01	0.9	6.8	4.5E−01	2.0	11.1	2.8E−01
	Shoulder abd + add	0.3	1.4	1.0E−01	0.4	1.9	1.8E−01	0.1	0.8	3.2E−01
	Shoulder int rot + ext rot	2.7	18.1	2.1E−01	3.8	15.2	1.5E−01	1.7	20.6	6.2E−01
	Shoulder hori flx + hori ext	−1.0	12.6	5.1E−01	−1.2	13.5	6.1E−01	−0.8	11.9	6.8E−01
	Elbow flx + ext	1.3	6.0	6.3E−02	0.9	6.3	4.2E−01	1.8	5.7	6.8E−02
	Wrist flx + ext	−0.8	10.3	5.3E−01	−0.6	8.2	6.8E−01	−0.9	12.0	6.3E−01
	Fingers V MCP flx + ext	1.3	15.1	4.8E−01	2.8	15.2	3.0E−01	−0.1	15.0	9.7E−01
Lower	Hip flx + ext	0.8	7.7	1.8E−01	0.3	8.1	8.3E−01	1.3	7.5	1.1E−01
	Hip abd + add	0.3	13.7	9.0E−01	2.0	11.0	3.1E−01	−1.2	15.7	6.1E−01
	Hip int rot + ext rot	2.3	12.0	8.3E−02	2.6	10.8	1.8E−01	2.1	13.0	2.5E−01
	Knee flx + ext	0.6	6.7	2.6E−01	−0.8	7.5	5.5E−01	1.9	5.8	*1.7E−02*
	Ankle dorsi flx + plantar flx	3.5	8.3	*2.9E−04*	3.8	8.0	*1.1E−02*	3.2	8.7	*1.1E−02*

Non-dominant (ND) and dominant (D) sides were determined by hand/arm for upper body and by foot/leg for lower body. *P* values less than 0.05 are shown in italics

*flx* flexion, *ext* extension, *abd* abduction, *add* adduction, *int rot* internal rotation, *ext rot* external rotation, *hori flx* horizontal flexion, *hori ext* horizontal extension, *dorsi flx* dorsi flexion, *plantar flx* plantar flexion

### Whole-body ROM patterns revealed by PCA

**Fig. 1:**
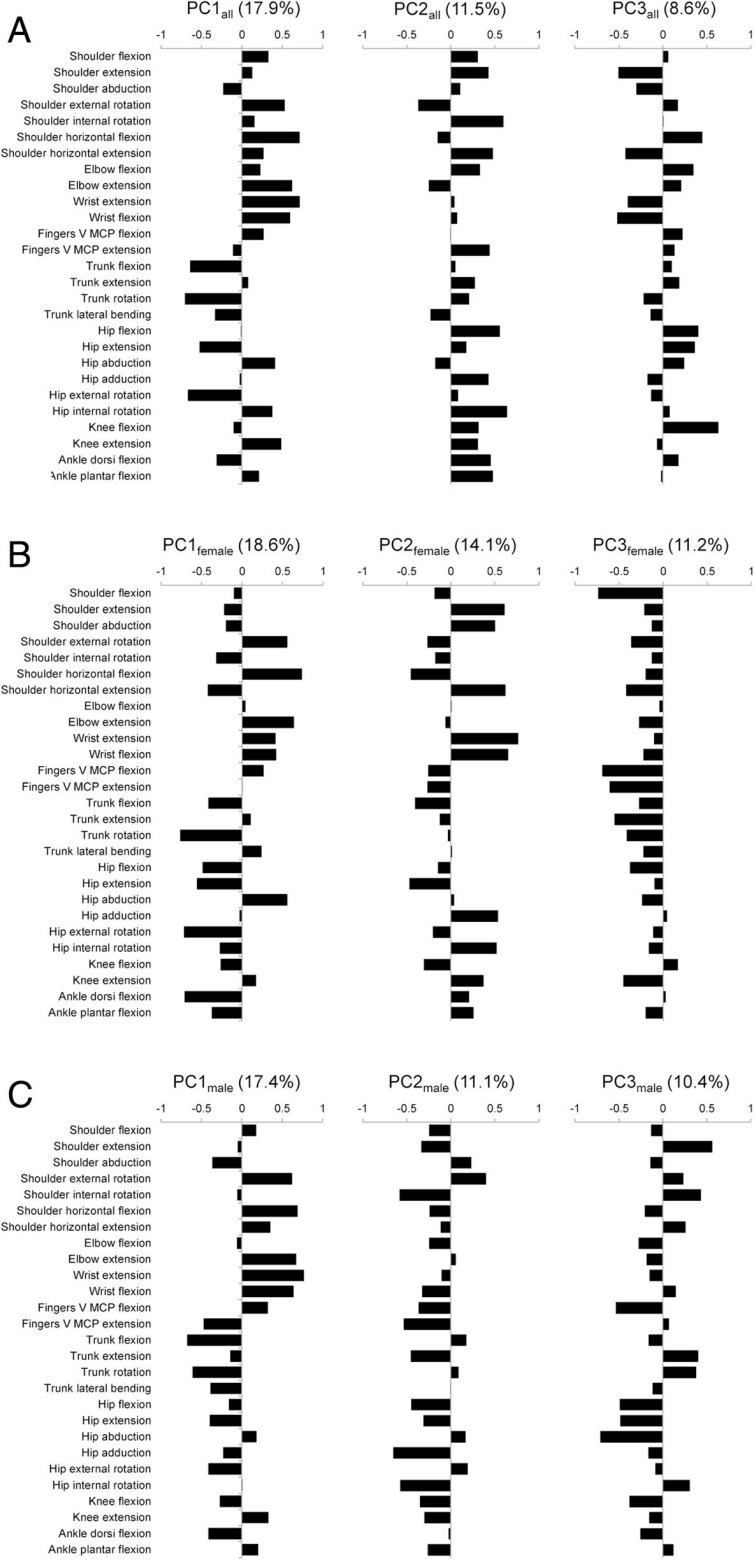
Principal component loadings of the first three PCs. **a** For all samples (females and males). **b** For female samples. **c** For male samples. The average of right and left ROMs was used when the joint motion had right and left data

**Fig. 2:**
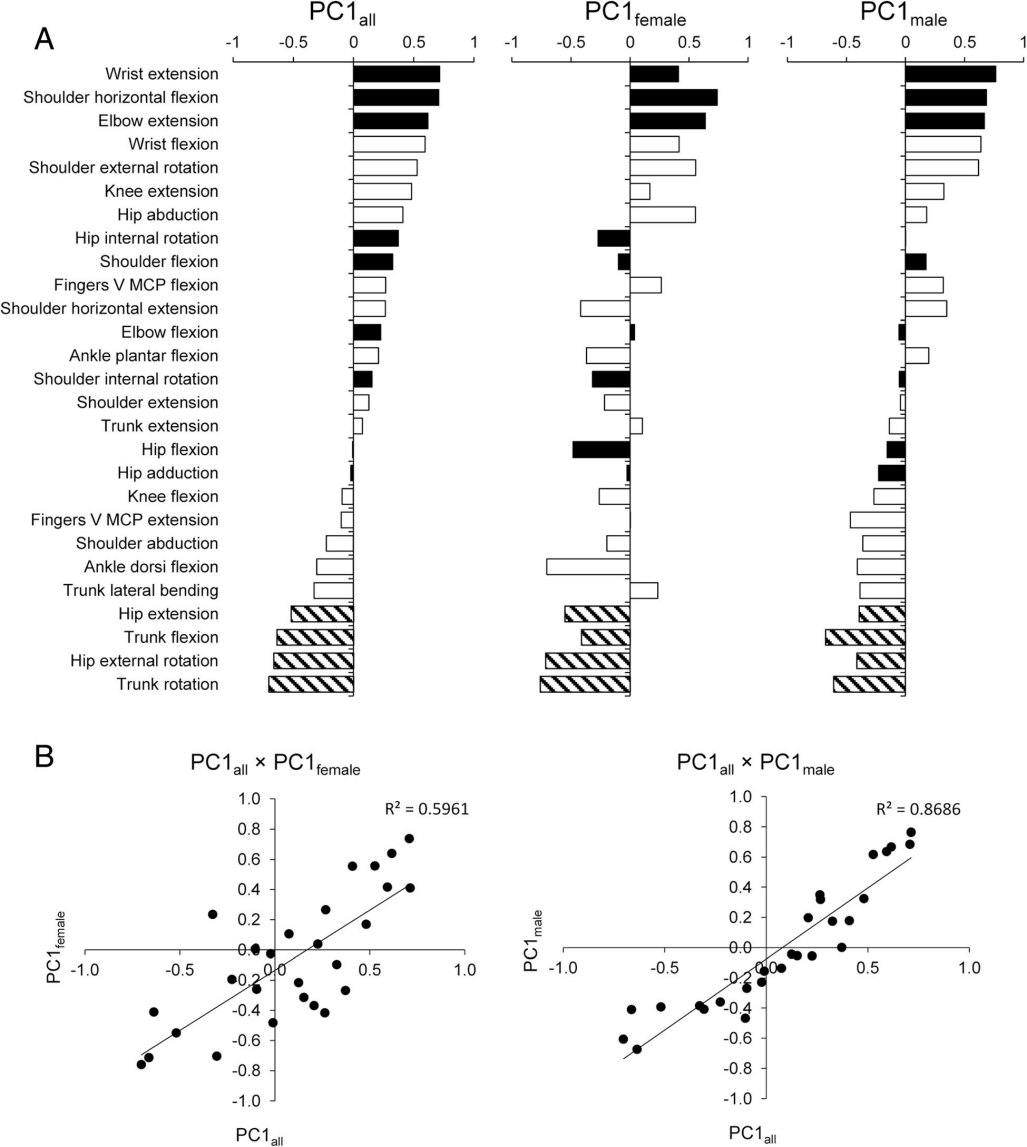
Comparison of PC1 in three PCAs using all samples, only female samples, and only male samples. **a** Principal component loadings. The joint motions are sorted in the order of loadings in PC1_all_. *Black bars* indicate motions in which females have a significantly larger ROM than males. *Shaded bars* indicate motions in which males have a significantly larger ROM than females. **b** Correlations between PC1_all_ and PC1_females_ and between PC1_all_ and PC1_males_

PCA was performed on the ROM data of all subjects, female subjects and male subjects. The first three PCs are shown in Fig. 1. When PCs resulting from female and male sample sets were compared, both sexes demonstrated similar patterns of PC loadings in PC1 (Fig. 2a), whereas different patterns were observed in PC2 and PC3. There were strong and significant correlations in the loadings between PC1_all_ and PC1_females_ (*r* = 0.75) and between PC1_all_ and PC1_males_ (*r* = 0.96) (Fig. 2b). Extremely positive values of loadings were observed for wrist extension, shoulder horizontal flexion, and elbow extension, and extremely negative values were observed for trunk rotation, hip external rotation, trunk flexion, and hip extension in PC1_all_. It is notable that the motions in which females have higher mobility than males showed positive values (black bars in Fig. 2a), whereas the motions in which females have lower mobility than males showed negative values (shaded bars in Fig. 2a). These results indicated that PC1_all_ is a component representing the sex difference, while this component is also observed within each sex.

### Factors associated with the whole-body ROM patterns

Table 5 shows correlation coefficients and partial correlation coefficients controlling for sex between PCs and the somatometric/sthenometric measurements. PC1_all_ was significantly correlated with age, BF%, iliospinale height, and leg extension strength after controlling for sex. PC2_all_ was also associated with sex, and significant partial correlations with age, forearm circumference, grip strength, leg extension strength, and leg flexion strength were observed. PC3_all_ was not affected by sex and had significant correlations with weight and forearm, minimum forearm, calf, and ankle circumferences.

**Table 5:** Correlations of PCs with somatometric/sthenometric measurements

Measurement	PC1_all_				PC2_all_				PC3_all_			
	*R*	*P*	R*	*P*	*R*	*P*	R*	*P*	*R*	*P*	R*	*P*
Sex	−0.51	*2.6E−06*	–	–	−0.45	*5.4E−05*	–	–	−0.16	1.7E−01	–	–
Age	−0.64	*4.5E−10*	−0.52	*2.7E−06*	0.23	*4.6E−02*	0.32	*7.4E−03*	0.07	5.7E−01	−0.01	9.1E−01
Height	−0.31	*6.0E−03*	0.23	5.8E−02	−0.31	*5.7E−03*	0.09	4.4E−01	−0.07	5.6E−01	0.04	7.6E−01
Weight	−0.36	*1.5E−03*	0.02	9.0E−01	−0.35	*1.8E−03*	0.12	3.1E−01	−0.23	*4.9E−02*	−0.14	2.5E−01
Body fat percentage	−0.10	3.7E−01	−0.48	*1.9E−05*	0.31	*6.4E−03*	0.15	2.1E−01	−0.15	2.0E−01	−0.12	3.2E−01
Lean body mass	−0.27	*2.0E−02*	0.23	5.6E−02	−0.40	*3.2E−04*	0.06	6.2E−01	−0.14	2.2E−01	−0.08	4.9E−01
Upper limb length	−0.35	*1.8E−03*	0.12	3.1E−01	−0.28	*1.5E−02*	0.12	3.3E−01	−0.04	7.2E−01	0.04	7.2E−01
Iliospinale height	−0.17	1.3E−01	0.36	*2.3E−03*	−0.32	*4.2E−03*	−0.05	6.8E−01	−0.08	5.0E−01	0.02	8.9E−01
Forearm circumference	−0.48	*1.2E−05*	−0.16	1.9E−01	−0.26	*2.2E−02*	0.28	*1.8E−02*	−0.24	*3.5E−02*	−0.17	1.6E−01
Minimum forearm circumference	−0.43	*1.0E−04*	−0.08	4.9E−01	−0.29	*1.1E−02*	0.15	2.0E−01	−0.24	*4.1E−02*	−0.10	4.1E−01
Calf circumference	−0.37	*9.6E−04*	−0.15	2.1E−01	−0.23	5.2E−02	0.17	1.5E−01	−0.27	*1.9E−02*	−0.17	1.5E−01
Ankle circumference	−0.14	2.3E−01	0.19	1.2E−01	−0.34	*2.7E−03*	−0.01	9.7E−01	−0.26	*2.6E−02*	−0.13	2.9E−01
Grip strength	−0.34	*2.8E−03*	0.14	2.4E−01	−0.15	2.0E−01	0.40	*5.4E−04*	−0.14	2.4E−01	−0.01	9.6E−01
Leg extension strength	−0.56	*2.1E−07*	−0.36	*1.8E−03*	−0.15	1.9E−01	0.31	*8.7E−03*	−0.20	9.2E−02	−0.13	2.8E−01
Leg flexion strength	−0.44	*8.7E−05*	−0.10	4.0E−01	−0.26	*2.6E−02*	0.24	*4.8E−02*	−0.12	3.3E−01	0.11	3.8E−01
2D:4D ratio	−0.04	7.5E−01	−0.07	5.8E−01	−0.04	7.4E−01	0.01	9.5E−01	−0.10	3.7E−01	0.02	8.4E−01

*P* values less than 0.05 are shown in italics

*R* correlation coefficient, R* partial correlation coefficient controlling for sex

To examine the independency of the effect of each factors and to further narrow down the factors that have a direct effect on the whole-body ROM pattern, we subsequently performed multiple regression analysis. Somatometric/sthenometric measurements that were significantly correlated with PCs as mentioned above were included as explanatory variables, and then the variables were chosen thorough stepwise procedures. As a result, sex, age, BF%, iliospinale height, and leg extension strength were associated with PC1_all_ (Table 6), which indicated that these factors have independent effects. PC2_all_ was associated negatively with being male but positively with age and grip strength. Grip strength was likely to represent the whole-body muscle strength since it was correlated with removed factors, leg extension, and flexion strength. The analysis for PC3_all_ suggested that the effects of weight and limb circumferences are not independent of each other, and forearm circumference could best explain the PC3_all_ scores.

**Table 6:** Multiple regression analysis for identifying factors that explain PCs

PC	Explanatory variables	*B*	*β*	*P*	Eliminated variables
PC1_all_	Sex (F:0, M:1)	−2.61	−0.60	*2.8E−06*	
	Age	−0.45	−0.28	*2.1E−03*	
	Body fat percentage	−0.14	−0.31	*5.4E−04*	
	Iliospinale height	0.13	0.35	*1.9E−03*	
	Leg extension strength	−0.02	−0.29	*2.0E−02*	
PC2_all_	Sex (F:0, M:1)	−3.20	−0.90	*3.7E−07*	Forearm circumference
	Age	0.35	0.27	*7.7E−03*	Leg extension strength
	Grip strength	0.11	0.58	*5.9E−04*	Leg flexion strength
PC3_all_	Forearm circumference	−0.19	−0.27	*1.8E−02*	Weight
					Minimum forearm circumference
					Calf circumference
					Ankle circumference

*B* partial regression coefficient, *β* standardized partial regression coefficient

P values less than 0.05 are shown in italics

## Discussion

The results for the sex difference for each ROM (Table 2) were mostly consistent with previous studies; the majority of limb joints had a larger ROM in females than in males, while males were more flexible than females in only four joint motions, including trunk flexion, trunk rotation, hip extension, and hip external rotation [14, 15, 18, 19]. The present study showed that age has negative correlations only with several joint motions. However, since ages of subjects were concentrated in early twenties, careful interpretation should be required for the effect of age. Because most individuals had a sports experience when they were high school students, the period of time after they ceased exercise may have an influence on ROM. Previous observations of a broader range of age groups have disclosed negative effects of age-related changes on the ROM patterns [2, 5, 8, 19]. A previous study of older adults showed that shoulder abduction and hip flexion are associated negatively with age and positively with muscular strength [26]; this may reflect that changes in physical activity due to ageing strongly affect both joint flexibility and muscular strength.

It is worth noting that the BF% and LBM showed not only negative effects on some joint motions but also positive effects on other joint motions. Negative correlations between BF% and several joint motions are likely due to physical obstruction by fat tissue caught between the bones constituting the joint. Shoulder horizontal flexion is a clear example of limitation by fat tissue (Table 2). The results of the multiple regression analyses indicated that the BF% contributes to the limitation of ROM in the upper limb, whereas it increases ROM in trunk flexion and rotation and hip external rotation. Causes of the positive correlation between BF% and ROM need to be further investigated. In the case of shoulder external rotation and horizontal flexion, effects of physical obstruction by muscles and the skeleton can explain the negative associations with LBM. On the other hand, the positive associations of LBM with wrist flexion and hip adduction can result from an indirect association; daily exercise may increase the flexibility of wrist and hip joints, as well as LBM.

Regarding hip joints, females were more flexible than males in flexion, adduction, and internal rotation, and vice versa in extension and external rotation (Table 2). However, no significant sex difference in the total range of antagonistic motions, such as flexion versus extension and external rotation versus internal rotation, suggests that each ROM of the hip joints is affected by skeletal morphology that determines relative positions and angles between bones. Sexual dimorphisms in anteversion of the acetabulum and femoral neck are well known; acetabular anteversion is defined as a forward tilt of the acetabular opening plane with respect to the sagittal plane, and femoral neck anteversion is defined as anterior rotation of femoral neck compared to the axis of the femoral condyles. In general, females have larger femoral neck anteversion than males, which is considered to be a reason for larger hip internal rotation and smaller hip external rotation in females than males [27, 28]. In addition, Nakahara et al. [29] found that a larger acetabulum anteversion in females than in males causes larger hip flexion and hip internal rotation, whereas males have larger ROMs than females in the antagonistic motions that are hip extension and hip external rotation.

As for the trunk, males had a greater ROM of flexion and rotation than females. Females generally have a shorter spinal column and a larger lumbar lordosis than males [30], which is considered to be a reason for females’ smaller trunk flexion and rotation. A kinematic analysis of rising from a chair reported that lumbar spine flexion occurs concurrently with hip flexion [31]; this suggests that lumbar spine flexion compensates for inflexibility of the hip joint motion in males.

The data on differences between dominant and non-dominant sides also provide information on factors affecting the variations in ROM. Joint motions that had a larger ROM on the dominant side than on the non-dominant side were shoulder external rotation, wrist flexion, and hip adduction. This result suggests an involvement of daily activity in the variation in ROM. Regarding the asymmetry of shoulder joints, it has been reported that the side of the dominant hand/arm has a significantly larger ROM than the other side, especially among individuals who have experience in sports with overhead-throwing motion [32, 33]. In the present study, we reanalysed only males who had experience in overhead-throwing motion sports, and confirmed an increased difference between the sides in shoulder external rotation (*n* = 19, ROM(ND) − ROMD) = −2.4 ± 4.2, *P* = 0.0245). On the other hand, some joint motions showed a larger ROM on the non-dominant side than on the dominant side. Of these motions, shoulder internal rotation and hip abduction are antagonistic movements of shoulder external rotation and hip adduction, respectively, that showed larger motion on the dominant side. These side differences may be due to off-centred neutral posture because the total ranges of antagonistic motions had no significant difference between the dominant and non-dominant sides. It is well known that side dominance causes asymmetry of posture. In addition, previous studies have reported that asymmetric daily posture, such as side sitting, can be related to ROM asymmetry [13, 34]. Alternatively, the difference between dominant and non-dominant sides may be attributed to muscle mass or extension of muscles and tendons; a forced and continuous motion on the dominant side increases its ROM by stretching the muscles antagonistic to the motion. In contrast, the reverse motion is limited by the developed muscles being an obstacle.

As for the relationships in ROM among different joints, Allander et al. [10] reported significant correlations among shoulder, wrist, metacarpophalangeal joint I (MCP I), and hip; in particular, wrist mobility was related to the mobility of the other three joints. However, no study has analysed the covariation patterns of whole-body ROM. On our PCA, PC1_all_ was associated with sex differences, and even when females and males were separately analysed, similar covariation patterns appeared as PC1. These results indicate that not only sexual dimorphism but also other factors, such as body fat, lower limb length, and muscle mass, can be involved in the component. In addition, our study also showed that PC2_all_ and PC3_all_ were significantly associated with muscle strength and limb circumference, respectively. This also indicated that body composition affects the whole-body patterns of joint flexibility.

**Fig. 3:**
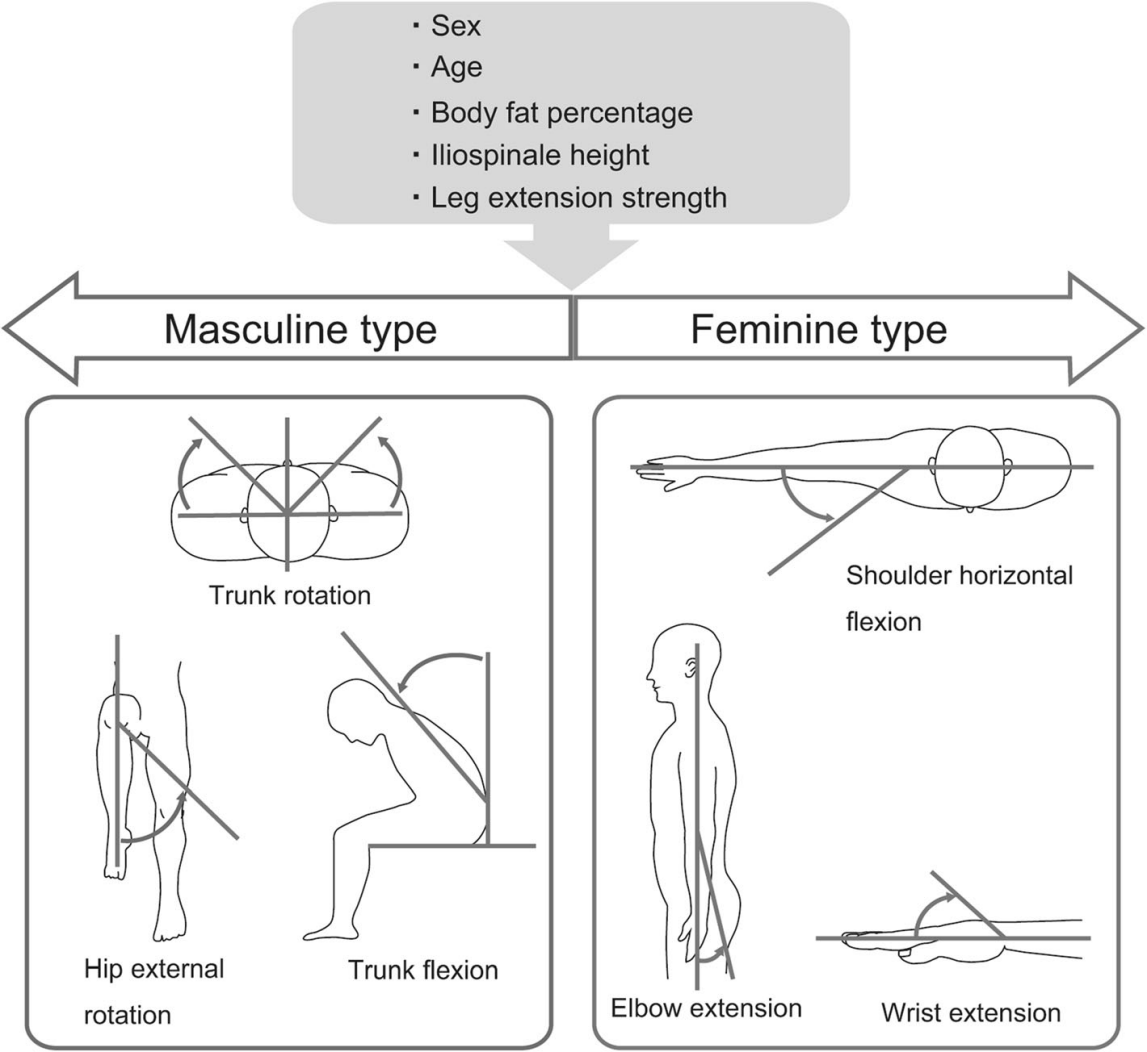
Schematic representation of the whole-body ROM patterns. Being flexible in some motions is characteristic of two contrary types, masculine type (*left*) and feminine type (*right*)

Based on the results of PCA, we refer to the positive direction of PC1_all_ as “feminine type”, and the negative direction as “masculine type” (Fig. 3). Feminine type is characterized by high flexibility of the upper limbs, such as wrist extension and flexion, shoulder horizontal flexion, and elbow extension, while masculine type is characterized by high flexibility of trunk flexion, trunk rotation, hip extension, and hip external rotation. On regression analysis, sex, age, BF%, iliospinale height, and leg extension strength were associated with PC1_all_. BF% had a negative association with the PC1_all_ score, which means that an increased BF% is related to masculine type. Golden et al. [35] have also suggested that an increase of BMI is correlated with a decrease of ROM and that a decreased amount of daily activity leads to both an increased BMI and decreased ROM in the whole body. Iliospinale height is an index of limb length; thus, the positive correlation between iliospinale height and PC1_all_ suggests that the longer the limbs are, the higher the tendency for feminine type the individual has. Leg extension strength, being an index of muscle mass, was associated with a tendency to have masculine type.

Furthermore, the covariation pattern of PC1_all_ should be strongly affected by acetabulum and femoral neck anteversion because antagonistic motions of hip joints, and hip abduction and adduction, were associated with sex oppositely. In a previous study, it was also reported that an increase in femoral neck anteversion contributed to a decrease in muscle strength of the gluteus medius and vastus medialis [36]. Therefore, the present study suggests that acetabular and femoral neck anteversion and muscle strength, being related to each other in a complicated manner, have an influence on hip joint ROM.

As shown above, multiple factors are likely to be associated with ROM and whole-body patterns of ROM. To understand how cultural differences affect ROM, further global comparisons will be indispensable [3, 10, 37]. In addition, genetic factors associated with joint flexibility still remain to be elucidated. A twin study has reported that the heritability for lumbar flexion is 64 % [38]. It has also been reported that the levels of femoral anteversion are highly correlated between siblings, indicating that this trait is partially heritable [39]. Therefore, the whole-body patterns of joint motions need to be further studied from the various perspectives, including genetic and environmental factors.

Our present study, clarifying the covariation patterns of joint flexibility, will contribute to the prevention of joint injuries and to the evaluation of dysfunction in patients with musculoskeletal diseases. For example, it has been known that anterior cruciate ligament injuries are more frequent in females than in males partly because of joint laxity [40], and therefore, it is possible that the “feminine type” has a higher susceptibility of the knee joint injury than the “masculine type” when they are compared within each sex. Further studies are needed to provide prevention and therapy programmes in consideration of the patterns of joint motions. In addition, it would be essential to know one’s type of joint flexibility and one’s suitable and effective postures and motions in order to improve the performance in sports and daily activities.

## Conclusion

A covariation pattern of ROMs that shows sexual dimorphism was found by PCA. Such covariation pattern was also observed within each sex as a spectrum of “masculine” and “feminine” types and was shown to be partly associated with body proportion and composition and with muscle mass and strength. Comparisons between dominant and non-dominant sides and between antagonistic motions provide suggestions that ordinary posture, daily motions, and skeletal morphology such as acetabular and femoral neck anteversion contribute to individual differences in ROMs. Such knowledge will contribute to the prevention of joint injuries and to improve one’s performance in sports and daily activities.

## Additional file

Additional file 1: Supplementary Tables. Table S1. Age, hand/arm and foot/leg dominances, and experience of sports of the subjects dominances of the subjects. Table S2. Somatometric and sthenometric measurements in the subjects. (DOCX 23 kb)

## Abbreviations

2D:4D: The ratio of the second and fourth finger lengths
AAOS: American Academy of Orthopaedic Surgeons
BF%: Body fat percentage
LBM: Lean body mass
MCP I: Metacarpophalangeal joint I
PCA: Principal component analysis
ROM: Range of motion
