# Supplementary material for: Whole-body patterns of the range of joint motion in young adults: masculine type and feminine type
Source: J Physiol Anthropol. 2016 Oct 1;35:23. doi: 10.1186/s40101-016-0112-8 (PMC5045662; doi:10.1186/s40101-016-0112-8)
Supplement: Additional file 1: Supplementary Tables. — Table S1. Age, hand/arm and foot/leg dominances, and experience of sports of the subjects dominances of the subjects. Table S2. Somatometric and sthenometric measurements in the subjects. (DOCX 23 kb) [file 40101_2016_112_MOESM1_ESM.docx]

**Supplementary Tables**

**Moromizato et al., Whole-body Patterns of the Range of Joint Motion in Young Adults: Masculine Type and Feminine Type**

Table S1. Age, hand/arm and foot/leg dominances, and experience of sports of the subjects

Table S2. Somatometric and sthenometric measurements in the subjects

Table S1. Age, hand/arm and foot/leg dominances, and experience of sports of the subjects

|  | Female | Male | Total |
| --- | --- | --- | --- |
| Number of subjects | 36 | 42 | 78 |
| Mean age　(±SD) | 20.8 (±1.2) | 21.4 (±1.9) | 21.1 (±1.6) |
| Age range | 20-25 | 20-29 | 20-29 |
| Right-handed | 30 | 35 | 65 |
| Left-handed | 4 | 2 | 6 |
| Both-handed | 2 | 5 | 7 |
| Right-footed | 34 | 34 | 68 |
| Left-footed | 1 | 6 | 7 |
| Both-footed | 1 | 2 | 3 |
| Experience of sports | 32 | 40 | 72 |
| Experience of overhead-throwing motion sports | 18 | 21 | 39 |

Table S2. Somatometric and sthenometric measurements in the subjects

| Body part | Item | All | | Female | | Male | |
| --- | --- | --- | --- | --- | --- | --- | --- |
|  |  | Mean | SD | Mean | SD | Mean | SD |
| Upper | Upper limb length [cm] (ND) | 70.7 | 4.5 | 67.1 | 2.9 | 73.9 | 3.1 |
|  | (D) | 70.8 | 4.4 | 67.4 | 3.0 | 73.8 | 3.2 |
|  | Forearm circumference [cm] (ND) | 23.0 | 2.1 | 21.4 | 1.3 | 24.4 | 1.5 |
|  | (D) | 23.5 | 2.2 | 21.8 | 1.3 | 25.1 | 1.6 |
|  | Minimum forearm circumference [cm] (ND) | 15.0 | 1.0 | 14.2 | 0.6 | 15.7 | 0.7 |
|  | (D) | 15.1 | 1.0 | 14.3 | 0.6 | 15.8 | 0.7 |
|  | Grip strength [kg] (ND) | 36.0 | 9.4 | 28.1 | 5.2 | 43.3 | 5.6 |
|  | (D) | 38.7 | 9.6 | 31.1 | 5.5 | 45.8 | 6.6 |
|  | 2D:4D ratio (ND) | 0.95 | 0.04 | 0.95 | 0.03 | 0.96 | 0.04 |
| Lower | Iliospinale height [cm] (ND) | 88.8 | 5.8 | 84.8 | 4.4 | 92.4 | 4.5 |
|  | (D) | 88.8 | 5.8 | 84.8 | 4.4 | 92.4 | 4.3 |
|  | Calf circumference [cm] (ND) | 34.2 | 2.1 | 33.0 | 1.6 | 35.2 | 2.0 |
|  | (D) | 34.0 | 2.2 | 32.9 | 1.6 | 34.9 | 2.2 |
|  | Ankle circumference [cm] (ND) | 20.3 | 1.2 | 19.6 | 1.0 | 20.8 | 1.0 |
|  | (D) | 20.3 | 1.2 | 19.6 | 1.0 | 20.9 | 1.0 |
|  | Leg extension strength [Nm] (ND) | 143.1 | 43.7 | 116.8 | 25.0 | 167.9 | 43.3 |
|  | (D) | 146.6 | 41.7 | 118.8 | 25.7 | 172.9 | 36.7 |
|  | Leg flexion strength [Nm] (ND) | 72.7 | 24.2 | 55.9 | 14.0 | 88.5 | 21.0 |
|  | (D) | 78.5 | 25.1 | 60.1 | 15.1 | 95.9 | 19.8 |

Non-dominant (ND) and dominant (D) sides were determined by hand/arm for upper body and by foot/leg for lower body.
